# Supplementary material for: Factors influencing healthcare workers’ and health system preparedness for the COVID-19 pandemic: A qualitative study in Ghana
Source: PLOS Glob Public Health. 2024 Jul 30;4(7):e0003356. doi: 10.1371/journal.pgph.0003356 (PMC11288451; doi:10.1371/journal.pgph.0003356)
Supplement: Attachment — Submitted filename: Response to reviewers.docx [file pgph.0003356.s004.docx]

Manuscript #PGPH-D-23-02021

Response to reviewer

Dear Dr. Cowley,

Thank you for allowing us to submit a revised draft of our manuscript entitled “Healthcare workers and health system pandemic preparedness: A qualitative study of COVID-19 in Ghana” for publication in *PLOS Global Public Health*. We are grateful for the time and effort that you and the reviewer, Dr. Kako, dedicated to our manuscript and are appreciative for the insightful remarks and valuable revisions. Those modifications are noted in the manuscript, and a point-by-point response to the reviewer’s comments and concerns is provided below in blue. All page and line numbers refer to the revised manuscript file with tracked changes.

Reviewer’s comments to the Authors

Thank you for your submission of this manuscript. I found that the manuscript will provide important insights of the HCWs in LICs where resource is scarce from non-disaster period. The manuscript will be improved with some additional information and clarifications.
some points for improvements.
1. Information, data clarification and presentations needs to be attended.
2. data collection methods need to be specified
3. some content clarifications needed some places.

Author response: Thank you! We have addressed your comments and concerns regarding clarity of data collection methods, presentation, results, and discussion in the revised manuscript.

Reviewer comment #1: I wonder the reasons to consider this study as a cross sectional study - this could be called as mixed method study? [pg. 6, lines 139-140]

Author response: Thank you for pointing this out. The reviewer is correct, and we have edited accordingly for clarity.

The revised text reads as follows:

“This qualitative study was embedded in a larger mixed-methods study of HCWs in Ghana conducted between April 17^th^, 2020 and March 8^th^, 2021” [pg. 5, lines 129-130].

Reviewer’s comment #2: Reviewer: I also wonder why you chose to interview those who are in managerial positioned people. If there is an intention the author should be explicit about this [pg. 6 line 150].

Author’s response: We appreciate the reviewer’s feedback. Given that health system leaders played a critical role in coordinating the COVID-19 pandemic response at the health facility level, we thought it was important to explore their perceptions of HCWs’ preparedness. We have specified our reasoning in the manuscript. The revised text reads as follows:

“In addition, we purposively sampled facility and health system leaders because they played a critical role in the COVID-19 response.” [pg. 6, lines 148-149]

Reviewer comments #3: How did you set up the interviewing - please specify the setting the interviewing what was the procedure for this? [pg. 6, line 159].

Author response:

We appreciate this feedback. We have clarified the methodology, as seen below in the revised text:

“Potential participants were first contacted by phone and given an overview of the study’s aims, objectives, and methods. Interviews were then scheduled later at a time convenient for the respondents.” [pg 6. Lines 149-151] […] All interviews were conducted in English over the phone by a researcher with qualitative training (FA), and lasted between 45 to 60 minutes.” [pg. 7, lines 165-167]

Reviewer comments #4: It is also helpful to insert the findings table to overview of this study [ pg. 8, line 192]

Author response: Thank you for bringing this to our attention. The overview of findings are presented in Table 1. Background characteristics of healthcare workers and administrators (pg. 8). Preparedness findings, in particular, are present at the end of the table. Nevertheless, we have made the presentation of these findings clearer by explicitly stating where the findings can be located:

“HCWs’ levels of preparedness are shown in Table 1.” [pg. 9, line 205]

Reviewer comments #5: think that this is another aspect of delaying and training [pg. 9, lines 216-217]

Author response: Thank you for pointing this out. We agree with the reviewer that this is another consequence of delayed training, which falls within the larger theme of inadequate training in preparing to handle the COVID-19 pandemic. For clarification, we have revised the text as follows:

“Inadequate COVID-19 pandemic preparedness was also attributed to inadequate training, particularly a lack of specialized training, as well as delayed training.” [pg.9, lines 216-217]

Reviewer’s comment #6: Please check the font

Author’s response: Thank you for pointing this out. The font has been corrected [pg. 14, line 361.

Reviewer’s comment #7: Please check writing style and format [pg. 15, line 377].

Author’s response. Thank you for noting this. We have corrected the format [ pg. 15, line 377].

Reviewer’s comment #8: Please check the sentence [pg. 16, line 416-418]

Author’s response: Thank you for noting this. The revised text reads as follows:

“HCWs recommended improving the clinical infrastructure, particularly by increasing the number of isolation centers, testing facilities, and equipment such as ventilators, to ensure health system and HCW readiness:” [pg. 16, lines 416-418]

Reviewer’s comment #9: Writing citation - please keep consistency of style [pg. 16, line 444]

Author’s response: Thank you for pointing this out. We have corrected the format and made sure the introduction of excerpts is consistent in style [ pg. 16, line 444].

Reviewer comment #10: especially in a case of CBERN disasters, it is evident [pg. 17, line 460].

Author’s response: Thank you for pointing this out. Although we agree with the reviewer, it is outside the scope of this manuscript because our investigation was solely focused on HCWs’ preparedness in the context of the COVID-19 pandemic.

Nevertheless, we have revised the text as follows for clarity:

“Our findings are concurrent with the broader literature on HCWs’ lack of preparedness to combat the COVID-19 pandemic.” [pg. 17, line 460]

Reviewer’s comment #11: I believe that this happened in many countries including HICs, but procurement [pg. 17, lines 467-469]

Author’s response: We agree with the reviewer’s assessment. We do, however, want to underscore that the healthcare supply chain shortfall disproportionately affected LMICS; thus, we have revised the text as follows:

“Though failure of the healthcare supply chain contributed to supply shortages worldwide, LMICs were disproportionately affected by a lack of resources, particularly reserves of PPEs (31).” [pg.17, lines 467-469]

Reviewer’s comment #12: I am not sure what you would like to say here it is not clear. [pg. 17, lines 517-520]

Author’s response: Thank you for pointing this out. We wanted to demonstrate the importance of capacity building in aiding HCWs’ preparedness. We have therefore revised the text as follows for clarity:

For example, a cross-sectional study in Saudi Arabia found that over 90% of HCWs who received training on safe and appropriate usage of PPE successfully passed the fit test for the N95 respirator mask and most demonstrated fair overall knowledge about the COVID-19 disease (37).”[pg.19, lines 517-520]

Reviewer’s comment #13: This global study comparison is out of blue and please make a point prior to write the global context would be helpful [pg. 20, line 556-560]

Author’s response: Thank you for pointing this out. We have accepted your later suggestion to improve the text for clarity. Therefore, we moved the last sentence in the paragraph before mention of the global study.

The text has been revised as follows:

“Therefore, it is probable that HCWs’ extensive management of earlier public health emergencies strengthened both their perceived confidence and preparedness to manage the COVID-19 pandemic. This is supported by findings from a cross-sectional study conducted in 57 countries, which showed that HCWs who had no prior outbreak experience scored significantly lower on measures of preparedness compared to HCWs who had prior experience with MERS, SARS, and avian flu outbreaks.(47).” [ pg. 20, lines 556-560]

Reviewer’s comment #14: I believe that this is a point that you would like to focus on. perhaps you can bring this sentence prior to the global context staring "a cross-sectional...." [ pg. 20, lines 556-560].

Author’s response: We think this is an excellent suggestion. We have therefore moved the last sentence in the paragraph to before mention of the global cross-sectional study. Please see the revised text below:

“Therefore, it is probable that HCWs’ extensive management of earlier public health emergencies strengthened both their perceived confidence and preparedness to manage the COVID-19 pandemic. This is supported by findings from a cross-sectional study conducted in 57 countries, which showed that HCWs who had no prior outbreak experience scored significantly lower on measures of preparedness compared to HCWs who had prior experience with MERS, SARS, and avian flu outbreaks.(47).” [ pg. 20, lines 556-560]

Reviewer’s comment #15: Please consider another conjunction [pg.20, line 565]

Author’s response: Thank you for this suggestion. We have changed the conjunction and have revised the text as follows:

“These findings are thus relevant to the early phase of the COVID-19 pandemic in Ghana and may not be reflective of later stages and subsequent improvements.” [pg.20, line 566]
