# Supplementary material for: Factors influencing healthcare workers’ and health system preparedness for the COVID-19 pandemic: A qualitative study in Ghana
Source: PLOS Glob Public Health. 2024 Jul 30;4(7):e0003356. doi: 10.1371/journal.pgph.0003356 (PMC11288451; doi:10.1371/journal.pgph.0003356)
Supplement: Attachment — Submitted filename: Author response 22March2024.docx [file pgph.0003356.s005.docx]

Manuscript #PGPH-D-23-02021R2

Response to reviewer

Dear Dr. Tucker,

Thank you for allowing us to submit a revised draft of our manuscript entitled “Healthcare workers and health system pandemic preparedness: A qualitative study of COVID-19 in Ghana” for publication in *PLOS Global Health*. We are grateful for the time and effort that you and the reviewers dedicated to our manuscript and are appreciative for the insightful remarks and valuable revisions. Those modifications are noted in the manuscript, and a point-by-point response to the reviewer’s comments and concerns is provided below in blue. All page and line numbers refer to the revised manuscript file with tracked changes.

| **COMMENTS** | **RESPONSE** | **Original Page/line no.** |
| --- | --- | --- |
| **Additional Editor/Reviewer Comments (if provided)** |  |  |
| Thank you for submitting your manuscript to PLOS Global Public Health. After careful consideration, we feel that it has merit but does not fully meet PLOS Global Public Health’s publication criteria as it currently stands. Therefore, we invite you to submit a revised version of the manuscript that addresses the points raised during the review process. Please pay particular attention to the comments from Reviewers 2 and 3, who both request additional details regarding the sampling methods, rationale, and the suitability of the survey to assess the study aims. Please clearly discuss the study limitations, such as the limited sample size and whether generalization of these findings across the country is valid. | Thank you! We have outlined the responses and revisions in response to the reviewers’ remarks below and on the corresponding pages of the article. |  |
| **Reviewer # 1 comments** | | |
| Thank you for your work on the comments. the revision of the manuscript addressed the respond of the comments and appropriately responded in the text. | Thank you very much! |  |
| **Reviewer # 2 comments** | | |
| Thank you for the opportunity to read this important work. The manuscript was very interesting and presents several key insights from the HCW perspective that will strengthen pandemic preparedness and response. I offer some suggestions the authors may consider: | Thank you for your review! We have outlined the responses and revisions in response to your remarks below and on the corresponding pages of the article. |  |
| The methods section would benefit from some brief elaboration on the qualitative approach taken - was this a qualitative descriptive study? Interpretative descriptive? A few sentences on the theoretical underpinnings of the work, or references, would be useful for the reader. | Thank you for pointing this out. We have clarified in the text that we used an exploratory qualitative descriptive research design and have cited references to support the theoretical basis of our approach.  The revised text reads as follows:  “The study used an exploratory descriptive qualitative research design to investigate HCWs’ perceptions of their own preparedness and that of the health system (30–32).”  We’ve also cited the following references in support of our qualitative research design:  Sandelowski M. What’s in a name? Qualitative description revisited. Res Nurs Health. 2010 Feb;33(1):77–84.  Chafe R. The Value of Qualitative Description in Health Services and Policy Research. Healthc Policy. 2017 Feb;12(3):12–8.  Doyle L, McCabe C, Keogh B, Brady A, McCann M. An overview of the qualitative descriptive design within nursing research. J Res Nurs. 2020 Aug;25(5):443–55. | p. 7., lines 181-182 |
| When presenting the overview of findings, it would be useful for the reader if the authors stated when the level of preparedness survey was conducted (at least the year). This would help the reader contextualize the snapshot that the survey provides within the overall timeline of the COVID-19 pandemic. | Thank you for noting this. We agree with your suggestion and have incorporated it into the manuscript. The revised text reads as follows:  “Table 1 shows the demographic information and other relevant quantitative data for the respondents in the qualitative arm of the study, taken from the survey data collected between April 2020 and May 2020.” | p. 8, lines 229-231 |
| Throughout the text it would be beneficial to put some text that highlights time and when during the pandemic the work was done. For example, it would be useful in the setting to put some time posts in - for example when the first case of COVID-19 was detected in Ghana, when COVID-19 vaccination began in Ghana etc - so that the reader gets a narrative sense of the arc of the pandemic in Ghana from first case to present day and where these interviews fit within that arc. This would also help draw out some key lessons learned from this work for future outbreaks or pandemics. | Thank you for pointing this out. The date of the first COVID-19 case in Ghana is noted under the Settings section. However, we have now included the date vaccinations were administered to the public.  The revised text reads as follows:  “COVID-19 vaccination began in Ghana on March 2, 2021 (24), with 32% of the population fully vaccinated as of July 2, 2023 (25).”- pg. 6, lines 152-153  Though we previously noted that the data was collected between April 17^th^, 2020 and March 8^th^, 2021, we have now added that:  “Thus, most of the data was collected in the early phase of the pandemic before vaccines were available in the country.” – pg. 6, lines 162-163 | p.6, lines 152-153; 162-163 |
| I wonder if the authors can do more in the discussion to move beyond the COVID-19 pandemic to make broader points about pandemic preparedness? While the retrospective focus of this work is interesting, I think the work could be strengthened by reflecting on these experiences from the perspective of 2024 and with a consideration of how the work presented here teaches us important lessons to prepare for the next pandemic. This is done a bit in the conclusion but could be done in the discussion as well to better frame the excellent points made in the final paragraph of the study. | We appreciate your suggestion. We have taken your suggestion into consideration and made broader points about pandemic preparedness beyond the COVID-19 pandemic throughout the discussion.  Please see the revised text below:  “The COVID-19 pandemic has underscored the necessity of creating an environment that supports HCWs to be sufficiently prepared to handle future pandemics, regardless of region or health sector (39).” – pg. 18, lines 505-507  “Ineffective management, lack of quality control of PPEs, and insufficient information about availability of PPEs remains an urgent public health concern, highlighting the need to address such issues to improve the health system’s preparedness for future pandemics (46–49).” – pg. 19, lines 522-525  “To prepare for future pandemics, HCWs need to receive sufficient training in pandemic disaster preparedness.”- pg. 20, lines 555-556  “It is thus imperative to critically assess present aid strategies as well as governance and coordination structures, and to define a new health-system strengthening approaches to prepare for future pandemics (39,57).” – pg. 20, lines 577-579. | p.18-20 |
| The text switches between ‘health care’ and ‘healthcare.’ I would suggest standardizing one or the other throughout. | Thank you for pointing this out. We have standardized the term “healthcare” throughout the manuscript. |  |
| **Reviewer # 3 comments** | | |
| The manuscript claims to have assessed healthcare workers and health system pandemic preparedness for COVID 19 in Ghana; however, with only 26 respondents interviewed who are responsible for the clinical management of patients and administrators at the health facility level, the study was not designed to provide a valid assessment of the state of Ghana’s health system preparedness. Moreover, further review of the manuscript reveals that it was more about the healthcare workers’ perception of how prepared they were to handle COVID 19 patients at the beginning of the pandemic. | Thank you for your comments. We acknowledge the concern about the numbers. The qualitative data presented here are part of a larger mixed-methods study in which we initially surveyed over 700 healthcare workers in Ghana. We chose a subset for the qualitative interviews since smaller samples are frequently used and generalizability is not necessarily the goal. With the 26, we reached data saturation, meaning that no new information was obtained after around 20 interviews. Thus, the sample size is appropriate for a qualitative study.  We also acknowledge that such data are insufficient to provide a comprehensive assessment of Ghana’s healthcare system preparedness. However, this is not the study’s purpose. Rather, we sought to explore HCWs and health systems’ leaders perceptions of factors that shaped their own preparedness as well as that of the health system for the COVID-19 pandemic. To clarify, we have now explicitly defined the purpose of the study at the conclusion of the introduction section. The revised text reads as follows:  “Our aim is to explore HCWs and health system leaders’ perceptions of factors that shaped their own preparedness as well as that of the health system.” – pg. 5, lines 140-141  We also acknowledge our title may have contributed to the confusion regarding the aims of the study; therefore, we have revised the title to better align with the aim of the study:  “Factors influencing healthcare workers’ and health system preparedness for the COVID-19 pandemic: A qualitative study in Ghana”  – Pg. 1, lines 2-3 | Pg. 1, lines 2-3  Pg. 5, lines 140-141 |
| Background  The authors did not provide a good context in previous studies/literature to build the groundwork for how to measure preparedness. Furthermore, the authors did not provide a sufficient background on the Ghanaian health system to allow the reader place the findings in perspective. Authors may wish to access this article by Oppenheim B, Gallivan M, Madhav NK, et al. for ideas on how to assess preparedness (Oppenheim B, Gallivan M, Madhav NK, et al. Assessing global preparedness for the next pandemic: development and application of an Epidemic Preparedness Index. BMJ Glob Health 2019;4:e001157. doi:10.1136/ bmjgh-2018-001157). | We have clarified that our aim is to explore HCWs’ and health system leaders’ perceptions of factors shaping their own and the health system’s preparedness, rather than to directly measure the health system’s preparedness. We believe that this clarification addresses this concern.  Thank you for sharing this reference. We reviewed it and observed that it concerns the development of an Epidemic Preparedness Index (EPI) for global comparisons, which falls beyond the scope of our study. It does, however, allude to the need for further contextual research to identify what factors may be contributing to key stakeholders’ perceptions of pandemic preparedness. We have cited it to support evidence of low preparedness in West Africa prior to the COVID-19 pandemic ( p. 4, lines 88-89; pg. 5, lines 113-114)  We have provided additional details about the Ghanaian health system to better contextualize the findings.  Please see the revised text below:  “The health system in Ghana has faced numerous challenges in responding to the pandemic. Ghana has a median of four ventilators and five staffed intensive care unit (ICU) beds, which are unequally distributed across the nation for a population of roughly 30 million (18,19). When the first COVID-19 cases were confirmed, 10 of the 16 administrative regions in the nation, home to a combined population of 10 million people, lacked an ICU bed (18,19). Even though Ghanaians’ health-seeking behaviors were intended to be improved by the introduction of the government-funded national insurance scheme, both public and private facilities have found it difficult to supply the essential resources for their operationalization due to lack of funding and poor budgetary allocation (19). For instance, on March 11, 2020, the president of Ghana announced an increase of $100 million to the health system (19,20). Though the funds were intended for equipment purchases, health education, and infrastructure, there were difficulties in allocating these funds and basic medical supplies like PPEs were not supplied to HCWs (19).” – pg. 5, lines 120-132 | Pg. 4, lines 88-89; pg. 5, lines 113-114; 120-132 |
| Methods  The details provided in the methodology are not sufficient to address the topic. It is also not clear how issues of bias and confounders were addressed. The sampling methodology employed is not clear. The authors explain that this was part of a survey designed to assess burnout and stress and therefore such a survey may not be designed to assess health systems preparedness. | We have clarified that this paper is not intended to evaluate the preparedness of Ghana’s health system. In the quantitative study, we examined healthcare workers’ perceptions of their preparedness, as well as burnout, stress, and job satisfaction. These publications are referenced in the manuscript.  In this qualitative study, we focus on their perspectives of the factors that influenced both their personal and health system’s preparedness. The methods of the quantitative study are detailed in depth in previous publications based on the quantitative data. In this paper, we merely briefly explain those methodologies before moving on to describe the qualitative study’s methods.  Because this is a qualitative study, we are unable to address concerns of confounding and bias associated with qualitative analysis. We did, however, take various efforts to improve the rigor of the qualitative analyses, including a team-based approach to coding and analysis that included diverse perspectives, as described in the methods.  We note that the qualitative sample was randomly drawn from participants in the quantitative study who provided their contact information to be contacted for a subsequent interview. We provided additional information on how they were contacted and interviewed under the qualitative sampling and data collection sections. |  |
| Results The methodology and data analysis do not fully support the claims made in the results section. It is not clear the criteria upon which the authors arrived at preparedness as presented in Table 1. Also Table 1 has family composition and it is not clear the relationship with preparedness. | We have clarified that the demographic information and other quantitative data presented in Table 1 are derived from the quantitative survey described in the section under parent study, as the revised text reads as follows:  “Table 1 shows the demographic information and other relevant quantitative data for the respondents in the qualitative arm of the study, taken from the survey data collected between April 2020 and May 2020.” Pg. 8, lines 229-231  We have provided more information from the quantitative study, including specifics on how perceived preparedness was measured. In the prior quantitative studies, we quantitatively examined the relationship between perceived preparedness and various demographic characteristics. In this qualitative study, the demographics are used solely to describe the qualitative sample.  The revised text reads as follows:  “Perceived preparedness was assessed in the quantitative study using a 15-item scale capturing personal, facility, and psychological preparedness for prevention, diagnoses, management, and education regarding COVID-19. Each question had response options ranging from 0 (not prepared at all) to 3 (very prepared). A perceived preparedness score was generated from the sum of responses to the 15 questions and categorized based on the mean score.” – pg. 6, lines 170-175 | Pg. 6, lines 170-175; pg. 8, lines 229-231 |
|  | Thank you for your review, which prompted us to clarify the study’s objectives. The paper is much improved now. |  |
